# Lack of intrafollicular memory CD4 + T cells is predictive of early clinical failure in newly diagnosed follicular lymphoma

**DOI:** 10.1038/s41408-021-00521-4

**Published:** 2021-07-15

**Authors:** Patrizia Mondello, Angelo Fama, Melissa C. Larson, Andrew L. Feldman, Jose C. Villasboas, Zhi-Zhang Yang, Ilia Galkin, Viktor Svelolkin, Ekaterina Postovalova, Alexander Bagaev, Pavel Ovcharov, Arina Varlamova, Sarah Huet, Bruno Tesson, Kaitlyn R. McGrath, Susan Slager, Brian K. Link, Sergei Syrbu, Anne J. Novak, Thomas M. Habermann, Thomas E. Witzig, Grzegorz S. Nowakowski, Gilles Salles, James R. Cerhan, Stephen M. Ansell

**Affiliations:** 1 Division of Hematology and Internal Medicine, Mayo Clinic, Rochester, MN USA; 2 Department of Medicine, Memorial Sloan Kettering Cancer Center, New York, NY USA; 3 Hematology Unit, Arcispedale Santa Maria Nuova, Azienda Unità Sanitaria Locale- IRCCS, Reggio Emilia, Italy; 4 Department of Health Sciences Research, Mayo Clinic, Rochester, MN USA; 5 Division of Hematopathology, Mayo Clinic, Rochester, MN USA; 6 BostonGene, Waltham, MA USA; 7 Hospices Civils de Lyon, Centre Hospitalier Lyon Sud, laboratoire d’hématologie, Pierre-Bénite, France; 8 Université Claude Bernard Lyon I, Lyon, France; 9 Institut Carnot CALYM, Paris, France; 10 Division of Hematology, Oncology and Bone Marrow Transplantation, University of Iowa, Iowa City, IA USA; 11 Department of Pathology, University of Iowa, Iowa City, IA USA; 12 Hospices Civils de Lyon, Centre Hospitalier Lyon Sud, service d’Hématologie, Pierre-Bénite, France

**Keywords:** Cancer microenvironment, CD4-positive T cells

## Abstract

Despite a characteristic indolent course, a substantial subset of follicular lymphoma (FL) patients has an early relapse with a poor outcome. Cells in the microenvironment may be a key contributor to treatment failure. We used a discovery and validation study design to identify microenvironmental determinants of early failure and then integrated these results into the FLIPI. In total, 496 newly diagnosed FL grade 1–3 A patients who were prospectively enrolled into the MER cohort from 2002 to 2012 were evaluated. Tissue microarrays were stained for CD4, CD8, FOXP3, CD32b, CD14, CD68, CD70, SIRP-α, TIM3, PD-1, and PD-L1. Early failure was defined as failing to achieve event-free survival at 24 months (EFS24) in immunochemotherapy-treated patients and EFS12 in all others. CyTOF and CODEX analysis were performed to characterize intratumoral immunophenotypes. Lack of intrafollicular CD4 expression was the only predictor of early failure that replicated with a pooled OR 2.37 (95%CI 1.48–3.79). We next developed a bio-clinical risk model (BioFLIPI), where lack of CD4 intrafollicular expression moved patients up one FLIPI risk group, adding a new fourth high-risk group. Compared with BioFLIPI score of 1, patients with a score of 2 (OR 2.17; 95% CI 1.08–4.69), 3 (OR 3.53; 95% CI 1.78–7.54), and 4 (OR 8.92; 95% CI 4.00–21.1) had increasing risk of early failure. The favorable intrafollicular CD4 T cells were identified as activated central memory T cells, whose prognostic value was independent from genetic features. In conclusion, lack of intrafollicular CD4 expression predicts early failure in FL and combined with FLIPI improves identification of high-risk patients; however, independent validation is warranted.

## Introduction

Follicular lymphoma (FL) is the most common form of indolent non-Hodgkin lymphoma, with an estimated 14,000 new cases diagnosed in the United States in 2016 [1]. Despite a typically indolent disease course, FL is clinically heterogeneous and some patients may progress early [2] or transform into an aggressive lymphoma, usually diffuse large B-cell lymphoma (DLBCL), with a poor outcome [3]. Early failure, defined as failing to achieve event-free survival at 24 months after diagnosis (EFS24) for patients treated with immunochemotherapy (IC) or failing to achieve EFS at 12 months (EFS12) for patients who were observed or received other treatments, is associated with an inferior overall survival (OS) compared with relapse after these landmarks, while patients achieving these landmarks have subsequent mortality equivalent to the background population [4]. Clinical prognostic scoring systems such as the Follicular Lymphoma International Prognostic Index (FLIPI) can be used to assess patient risk and predict the outcome [5]. This index uses standard clinical and laboratory findings that are surrogates of the underlying disease. However, the FLIPI does not predict which patients will fail EFS12/EFS24, hence, it is not routinely used to guide therapeutic decisions [4]. Several efforts to identify biologic and genetic factors that predict survival or risk of transformation have been attempted over the years [6,7,], but no definitive biomarkers have found routine application in clinical practice to date.

Gene expression profiling studies of FL have demonstrated an association between immune cells in the tumor microenvironment and disease progression and survival [8]. Several immunohistochemical studies attempted to correlate specific T-cell subsets with outcome; however, mixed and inconclusive results have been reported [9–13]. One explanation of this discrepancy might be the evaluation of the total expression of immunohistochemical markers throughout the malignant lymph node, rather than intra- or perifollicular biomarker expression. In particular, the immune architectural pattern of T cells within lymph nodes involved with FL has a strong clinical impact since the topographic distribution of the immune cells reflects their dysregulated function [11]. Based on this assumption, we hypothesized that the phenotype and distribution of cells in the tumor microenvironment would predict early failure, and that integrating these data into clinical prognostic models would improve risk stratification for patients with FL. To address this hypothesis, we analyzed the prevalence of T-cell subsets and macrophages in the pretreatment biopsy specimens of newly diagnosed patients with FL who were prospectively enrolled in the Molecular Epidemiology Resource (MER) cohort at the Mayo Clinic and University of Iowa. To explore the potential relevance of the interactions between immune cells and malignant B cells, we specifically determined the expression of immune markers inside and outside the malignant follicles. Immunohistochemistry (IHC) was performed on a discovery cohort and promising findings were then tested in a validation cohort. The prognostic value of the IHC findings was evaluated alone and then in combination with FLIPI. For biological validation, the immune population associated with outcome was immunophenotypically characterized and spatially located in the follicle using mass cytometry (CyTOF) and the Co-Detection by indEXing (CODEX) multiplex immunofluorescence system. Finally, the influence of the tumor genetic landscape on the microenvironment was assessed by a digital multiplex gene expression profiling (NanoString technology) platform on the same matched patients [7].

## Materials and methods

For detailed methods on IHC, CyTOF, and CODEX analysis, please refer to the Supplementary Materials.

### Patients

Patients with newly diagnosed FL grade 1–3 A were prospectively enrolled from 2002 to 2012 into the University of Iowa/Mayo Clinic SPORE MER cohort study [14]. Histologic diagnosis was performed according to World Health Organization criteria by an expert pathologist at each participating center [15]. Availability of sufficient biopsy tissue at diagnosis to obtain two 0.5-mm cores for incorporation into a tissue microarray (TMA) was the only inclusion criteria for this study. The discrimination between discovery and validation cohorts was based on tissue availability at different time points of the study. Specifically, 166 TMAs were used for the extensive screening and subsequent 330 for validation of promising results. The initiation of treatment was guided by the Groupe d’Etude des Lymphomes Folliculaires (GELF), BNLI or National Comprehensive Cancer Network (NCCN) criteria [16–18]. All patients were followed for disease progression/relapse, retreatment, and death; and all events were validated with medical records. The study was reviewed and approved by the human subjects’ Institutional Review Board at the Mayo Clinic and at the University of Iowa, and written informed consent was obtained from all participants.

### Statistical analysis

EFS was defined as time from diagnosis to progression, relapse, retreatment, or death due to any cause. Early failure was defined as failing to achieve EFS at 24 months (EFS24) for patients treated with IC at diagnosis, and failing to achieve EFS at 12 months (EFS12) for patients who were observed or received other treatments, with EFS12/24 referring to the combined endpoint of disease progression when analyzing all patients [4]. The association of the IHC markers with risk of early failure was estimated using odds ratios (ORs) and 95% confidence intervals (CI) from logistic regression models. Markers at *P* ≤ 0.15 were brought forward for replication in a separate set of patients from the MER cohort (*N* = 330), with a similar OR and *P* < 0.008 (Bonferonni correction of 0.05/6 tests) being declared a statistically significant validation. In the discovery cohort the arbitrary p-value cut-off of *P* ≤ 0.15 was simply used to filter down promising biomarkers with no additional testing, while in the validation cohort, the results were corrected for multiple testing. In the combined dataset, we also used Cox regression to assess the associations with continuous EFS and OS. We next developed a bioclinical risk model for EFS, adding IHC-determined intrafollicular CD4 + cells to the 3-level FLIPI risk grouping (0–1, 2, and 3–5). We used FLIPI rather than FLIPI-2 [19] because β_2_-microglobulin serum concentrations are not routinely measured, particularly in patients diagnosed before the FLIPI-2 was described, and because the original FLIPI is more commonly used [20]. We compared the performance of the various models using c-statistics.

## Results

### Study populations

Between 2002 and 2012, 918 consecutive newly diagnosed patients with FL were enrolled into the MER. A total of 496 patients with available diagnostic biopsies on a TMA were included in this analysis, while 422 were excluded for lack or insufficient tissue. (Supplementary Fig. 1) To assess potential selection bias, we compared the characteristics of patients in the study with those excluded. There was not a significant difference between the two cohorts, except for the involvement >4 nodal sites (39.8% vs 29.7%, *p* = 0.002) and higher histological grade (FL3A 15.9% vs 9%, *p* = 0.002) (Supplementary Table 1). While this difference may in part explain the more frequent tissue availability, it does not imply substantial clinical dissimilarity. The discovery cohort consisted of 166 patients with a median age at the time of diagnosis of 60 years (range, 23–91). Most patients had stage III–IV disease (71.1%), grade 1–2 (83.1%) FL, normal LDH levels (80.3%), and low/intermediate FLIPI score (71.3%). One-third of patients received IC (37.3%), one-third were observed (33.1%), and the remainder received rituximab monotherapy (10.8%), radiotherapy alone (6.6%), or other treatments (12%). After a median follow-up of 11.3 years, 81.3% achieved EFS12/24 (98 events overall) and the median OS was 14.4 years (46 deaths overall) (Supplementary Table 2).

The validation cohort consisted of 330 patients with a median age of 58 years (range, 24–93). Overall, patient characteristics between the two cohorts were similar. After a median follow-up of 6.9 years, 76.7% achieved EFS12/24 (181 events overall) and the OS was not reached (64 deaths overall). (Supplementary Table 2)

### Intrafollicular CD4 + T cell expression is an independent prognostic factor in FL

In the discovery cohort of FL patients, we investigated the association between immune biomarker expression patterns (i.e., inside vs outside the follicle, Supplementary Fig. 2) and the risk of early failure. Out of 22 associations (11 biomarkers each scored for inside and outside the follicle), six were associated with early failure at p ≤ 0.15: (i) absence of CD4 + expression inside and (ii) outside the malignant follicles; (iii) absence of CD8 + expression inside the malignant follicles; (iv) absence of PD-1 (marker of T-follicular helper [T_FH_], CD8 + , T-regulatory [T_REG_]) expression inside the follicles; (v) absence of T_REG_ marker FOXP3 expression outside the follicles; (vi) expression of macrophage surface marker SIRPα inside the follicles (Supplementary Fig. 3A and Supplementary Table 3). Other macrophage markers, such as CD14 and CD68, the activated T-cell markers CD32b and CD70, and the T-cell exhaustion markers PD-L1 and TIM3, were not significantly associated with early failure (Supplementary Table 4).

**Fig. 1:**
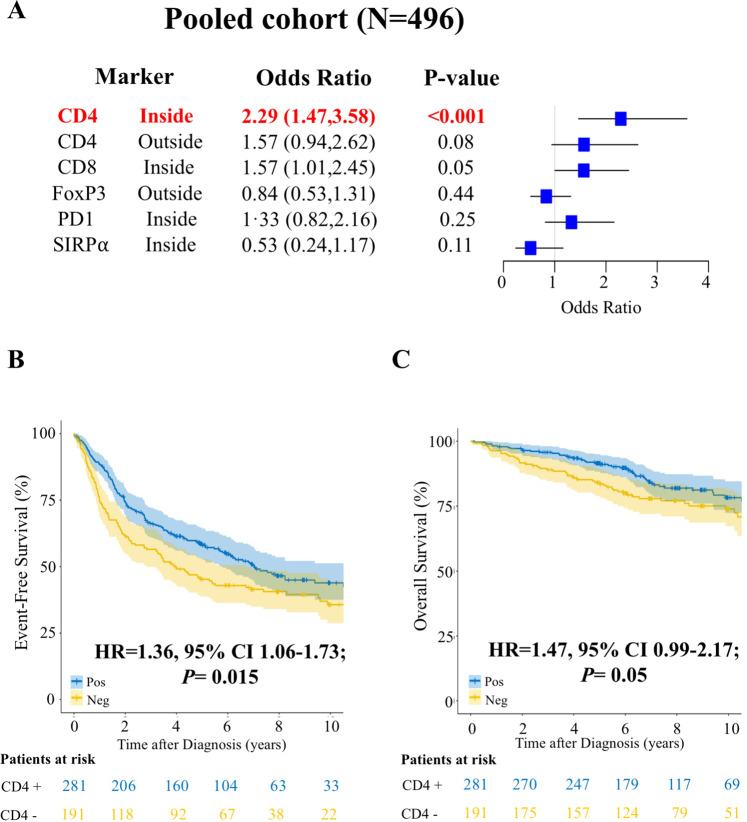
Absence of intrafollicular CD4 + expression is an independent prognostic factor in follicular lymphoma. **A** Forest plots showing the association between indicated immune biomarkers and early failure in the pooled cohort (*N* = 496) of follicular lymphoma patients. **B** and **C** Kaplan–Meier curves representing event-free survival (**B**) and overall survival (**C**) in the pooled cohort of follicular lymphoma patients with a positive (blue) or negative (yellow) intrafollicular CD4 + expression.

We then evaluated the six promising IHC markers in the validation cohort. Among all the previously significant T cells and macrophage markers, only CD4 + inside of the follicles was validated (Supplementary Fig. 3B and Supplementary Table 3). Specifically, lack of intrafollicular CD4 + expression was associated with higher risk of early failure in the discovery (OR 1.77, *p* = 0.15) and validation (OR 2.59, *p* = 0.001) cohorts, with a pooled risk of OR 2.29 (95% CI 1.47–3.58; *p* < 0.001) (Fig. 1A and Supplementary Table 3). In the pooled dataset, adjustment for FLIPI did not impact the association (OR 2.28; 95% CI 1.44–3.63; *p* < 0.001), and lack of CD4 + expression was associated with higher risk of early failure in FLIPI groups 0–1 (OR 2.65, 95% CI 1.07–6.80), 2 (OR 2.07, 95% CI 0.96–4.56) and 3–5 (OR 2.51, 95% CI 1.21–5.32). The prognostic value of CD4 + expression remained also after adjustment for treatment groups with OR 2.20 in IC (95% CI 1.09–4.45) vs OR 2.53 in non-IC (95% CI 1.33–4.83). We then explored whether CD4 + expression holds prognostic utility in patients receiving different chemotherapy backbones. However, its significance was lost when adjusting for anthracycline-based (OR 2.35, 95% CI 0.83–6.63) vs non-anthracycline-based (OR 2.21, 95% CI 0.80–6.13) regimens likely due to the low power of each subpopulation. Lack of intrafollicular CD4 + expression was significantly associated with inferior continuous EFS (HR = 1.36, 95% CI 1.06–1.73; *p* = 0.015; Fig. 1B) and OS (HR 1.47, 95% CI 0.99–2.17; *p* = 0.05; Fig. 1C), and these associations held after adjustment for FLIPI (HR 1.31, 95% CI 1.03–1.68; *p* = 0.03, and HR 1.44, 95% CI 0.97–2.12; *p* = 0.07, respectively).

### Integrating intrafollicular CD4 + expression into the FLIPI score improves risk stratification of FL patients

**Fig. 2:**
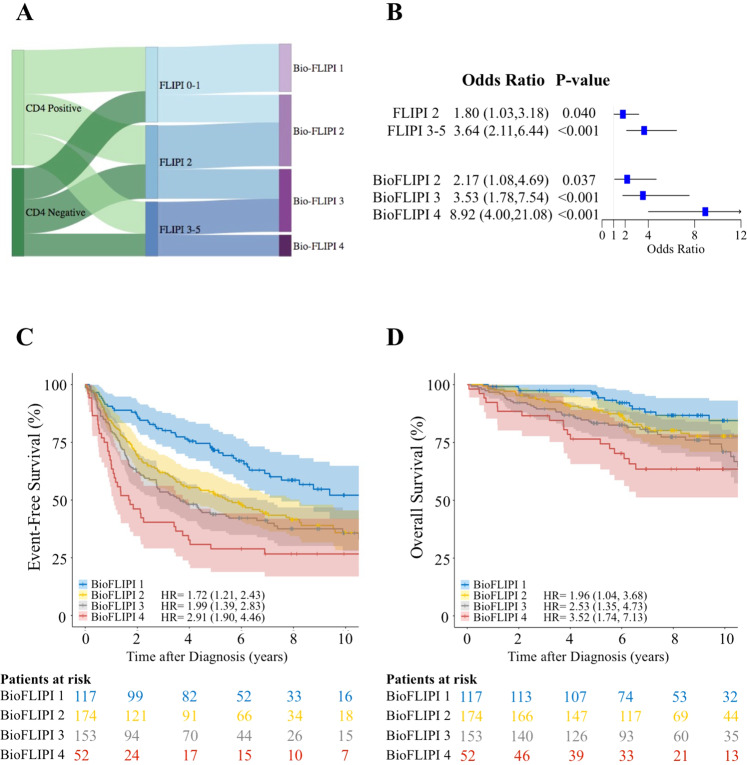
The BioFLIPI is an improved prognostic risk model. **A** Distribution of the FLIPI subgroups within CD4 + expression (left) and BioFLIPI risk groups (right). **B** Forest plots showing the association between FLIPI and BioFLIPI risk groups and early failure in the pooled cohort (*N* = 496) of follicular lymphoma patients. **C** and **D** Kaplan–Meier curves representing event free survival (**C**) and overall survival (**D**) in the pooled cohort of follicular lymphoma patients classified by BioFLIPI risk groups.

Given the independence of intrafollicular CD4 + expression in predicting prognosis, we hypothesized that integrating this biological factor into the well-established clinical prognostic model FLIPI would improve risk stratification of FL patients and ultimately help in guiding therapeutic decisions. We established a bioclinical risk model termed BioFLIPI that combined the intrafollicular CD4 + expression and FLIPI into a 1–4 scale, where lack of intrafollicular CD4 + expression moved a patient up one FLIPI risk group, adding a fourth risk group for FLIPI 3–5 and the absence of intrafollicular CD4 + expression. This new classification moved about 40% in each FLIPI risk group to the next higher BioFLIPI group, with 10% of the overall cohort in the new highest-risk group (Supplementary Table 5 and Fig. 2A).

BioFLIPI was a better predictor of early failure than FLIPI. Compared with a BioFLIPI score of 1 (24% of patients), patients with a score of 2 (OR 2.17, 95% CI 1.08–4.69), 3 (OR 3.53, 95% CI 1.78–7.54), and 4 (OR 8.92, 95% CI 4.00–21.1) had an increasing risk of early failure (Fig. 2B). The c-statistic for BioFLIPI (0.665) was slightly higher than that for FLIPI alone (0.636). Similarly, the BioFLIPI better predicted EFS as a continuous variable (Fig. 2C and Supplementary Fig. 4A) and overall survival (Fig. 2D and Supplementary Fig. 4B). This prognostic significance was maintained in analyses stratified on initial treatment with immunochemotherapy vs. not (Supplementary Fig. 5A–D and Supplementary Table 6). Taken together, these data suggest that BioFLIPI is a better predictor than FLIPI, but it requires further validation in an independent population.

### Intrafollicular activated, nonexhausted, central memory T cells display a crucial role in the microenvironment of FL

**Fig. 3:**
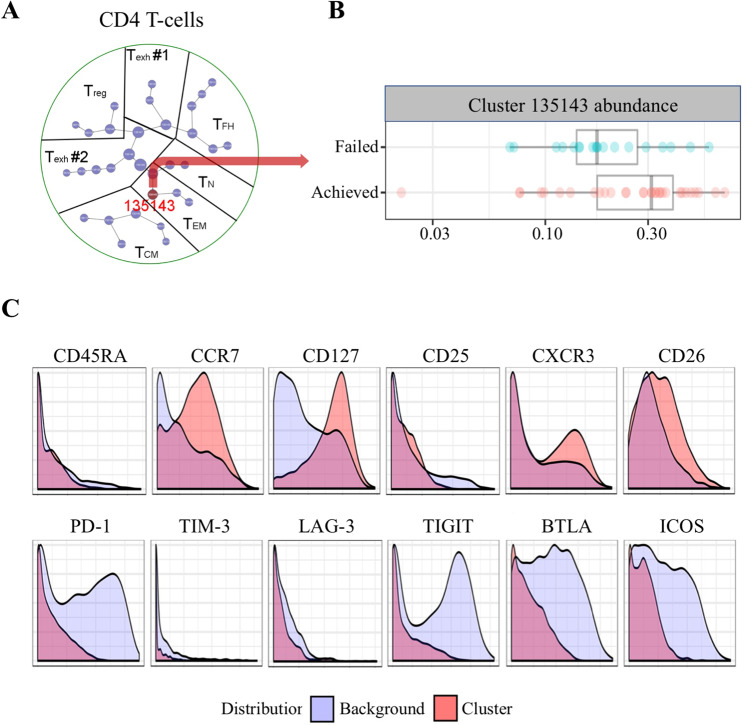
Activated, nonexhausted central memory T cells play a crucial role in follicular lymphoma. **A** Plot showing hierarchical clustering of CD4 + T cells from 51 newly diagnosed FL patients who achieved (*N* = 32) vs failed (*N* = 19) EFS12/24. The red circle represents the cluster that differed between the two groups. Number in the circle indicates the cluster ID. **B** Graph showing the difference in cells abundance in cluster 135143 between patients who achieved (*N* = 32) vs failed (*N* = 19) EFS12/24. **C** Histogram plots showing the expression of indicated cell markers from the parent cluster (red) over the background (light blue) in cells from cluster 135143.

To better characterize the T cell phenotype of CD4 + immune cells that are associated with outcome in FL, we performed CyTOF on viable, cryopreserved, single-cell suspensions from tumor biopsies of 51 newly diagnosed FL patients from our cohort (Supplementary Fig. 6 and Supplementary Table 7). We selected CD4 + T cells from the FL specimens and performed clustering analysis using the software Cluster 3.0. To explore whether this clustering had clinical relevance, we then performed a CITRUS analysis to identify significant clusters between patients who achieved or failed EFS12/24 (32 and 19 patients, respectively). We found that only one cluster of CD4 + T cells, cluster 135143, significantly differed between the two patient groups (Fig. 3A, B). The cluster 135143 was defined by a distinct CD45RA– CCR7 + T cell phenotype, suggesting a central memory T cell (T_CM_) type as opposed to CD45RA–CCR7-effector memory T cells (T_EM_) [21]. Interestingly, the CD4 + CD45RA–CCR7 + T cells displayed high expression of CD26, CD127, and CXCR3, indicating an activated T_CM_ phenotype. In contrast, these CD4 + T cells did not express T_FH_ markers, including PD-1 and inducible T-cell costimulator (ICOS), nor T-cell exhaustion markers, such as TIM3, LAG-3, TIGIT, and BTLA (Fig. 3C). However, our panel did not include CXCR5, which might have had provided further information on T_FH_ cells.

**Fig. 4:**
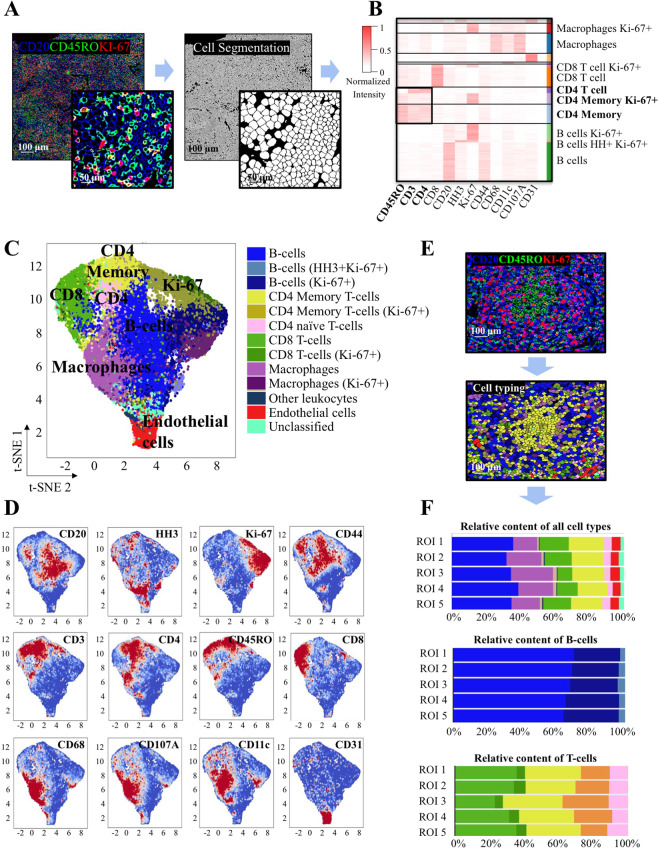
Tumor characterization of follicular lymphoma. **A** Representative images showing the correspondence between composite fluorescence and cell segmentation. **B** Heatmap showing marker expression within each identified cell cluster. Values are depicted in a colorimetric scale from red (high) to white (low). **C** *t*-SNE plot of cell clusters derived from 5 regions of interest (ROI). **D** *t*-SNE plots showing expression of indicated markers from **C**. Values are depicted in a colorimetric scale from red (high) to blue (low). **E** Representative images showing that intrafollicular CD20-CD4 + CD45RO + cells correspond to CD4 + memory T cells. **F** Bar graphs showing similar frequency of the indicated cell subsets among the five ROIs.

Since the CyTOF assay included all the cells in the tumor without discriminating intra- vs. perifollicular T cells, we performed CODEX analysis to provide spatial information on the immune architectural pattern of the lymph node and validate the CyTOF findings. Multiplexed images generated by the CODEX system underwent single-cell segmentation using a machine-learning approach (Fig. 4A, Supplementary Table 8 and Supplementary Fig. 7A–E). Single-cell events were extracted, including marker intensity expression and spatial localization, and underwent unsupervised clustering followed by manual merging and annotation of cell types (Fig. 4B). We identified 14 unique groups of defined cell subsets (12 immune cell clusters, one vascular and one undefined cluster). The phenotypic profile of annotated cell types was inspected using a dimensionality reduction tool (*t*-SNE) (Fig. 4C, D). The CD3 + CD8 + T cells were mostly located outside the follicles (Supplementary Fig. 8A), whereas the CD3 + CD4 + T cells were found both inside and outside the follicles. Notably, the majority of CD3 + CD4 + T cells inside the follicles coexpressed CD45RO + and were identified as CD4 + memory T cells, while most of the CD3 + CD4 + T cells outside the follicles did not express CD45RO and were classified as non-memory CD4 + T cells (Fig. 4E and Supplementary Fig. 8B,C). The relative frequencies of each cell type within the five imaged follicular regions were calculated and appeared similar among regions (Fig. 4F).

**Fig. 5:**
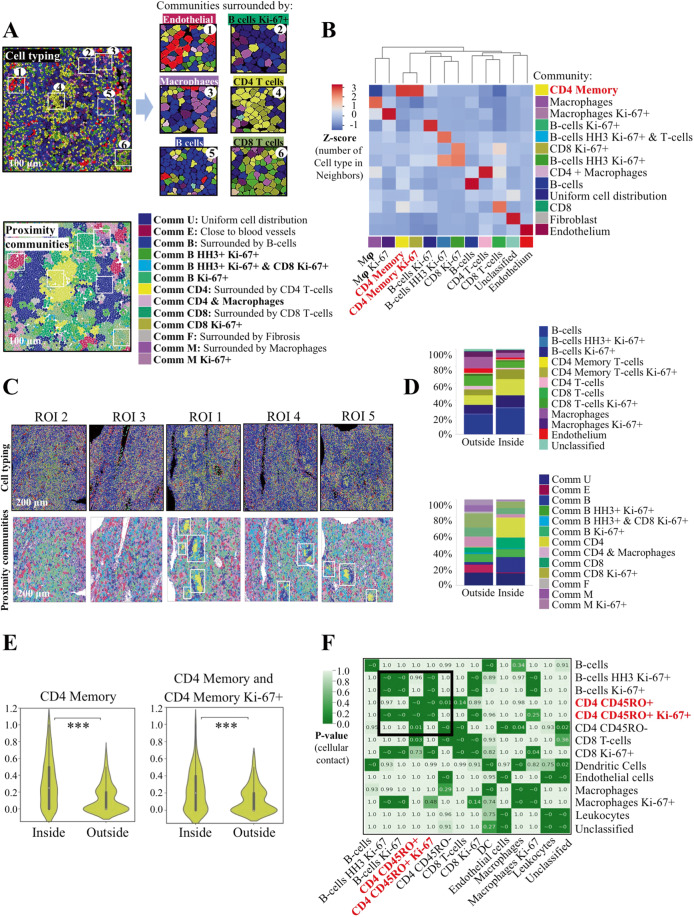
CD4 + memory T cells are prevalent inside the follicles. **A** Representation of cell contours from the neural network showing cell types (top) and community (bottom) identification. **B** Heatmap showing the average number of selected cell types as neighbors for cells from the community. Scaled values (z-scores) are depicted in a colorimetric scale from red (high) to blue (low). Mφ, macrophages. **C** Representative images showing cell types and community identification in five ROIs. **D** Bar graphs showing the percentage of cell types and communities inside and outside the follicles from (**C**). **E** Violin plots showing the increased frequency of CD4 + memory or CD4 + memory:CD4 + memory Ki-67+ interactions inside compared with outside the follicles in five ROIs. **F** Heatmap showing the significance of neighboring contacts between each pair of cell types from (**C**). *P*-values are depicted in a colorimetric scale from light green (low) to dark green (high). DC, dendritic cells.

To take a deeper look into the spatial organization of tumor cells, immune cells, and other microenvironment components, we reasoned that the dynamic spatial contexts of the tissue could be recapitulated using a spatial analysis approach. In this strategy, first cell-to-cell contacts between the different identified subsets were calculated from single-cell segmented images. Subsequently, cellular communities (or neighborhoods) were identified by clustering cells based on the number and type of contacts (neighbors). Given that cells might exist in multiple neighborhoods simultaneously or cell neighborhoods might overlap, every cell type was assigned to a single community to simplify visualization and interpretation of the spatial behavior of the tissue. We found 13 communities that recapitulated the core tissue components, as validated on the original fluorescent images (Fig. 5A and Supplementary Fig. 9A, B). Notably, the community surrounded by CD4 + memory T cells was the only one enriched for CD4 + memory T cells and activated CD4 + memory T cells (Fig. 5B). We then evaluated the cell types and proximity communities relative to follicular distribution and found that the rate of CD4 + memory T-cells was greater inside the follicles compared with outside the follicles (20.4% vs 11.2%, *p* < 0.001, Fig. 5C, D). Accordingly, the community of CD4 + T cells was prevalent inside the follicles (26.3 ± 4% vs 0.004%, *p* < 0.001, Fig. 5C, D). The frequency of contacts between CD4 + memory T cells reciprocally and between CD4 + memory T cells and CD4 + memory T-cell Ki-67+ was significantly increased inside the follicles (*p* < 0.001, Fig. 5E). On a single-cell level, the contacts between CD4 + memory T cells and CD4 + memory T-cell Ki-67+ were found to be non-random (Fig. 5F). Collectively, these data demonstrate that the intrafollicular CD4 + T cells that drive outcome in FL are activated, nonexhausted effector memory T cells (Supplementary Fig. 10), a subset that is well-known to play a critical role in the immune response against the tumor [22].

### Intrafollicular CD4 + T cells and tumor gene expression profile independently predict outcome in FL

**Fig. 6:**
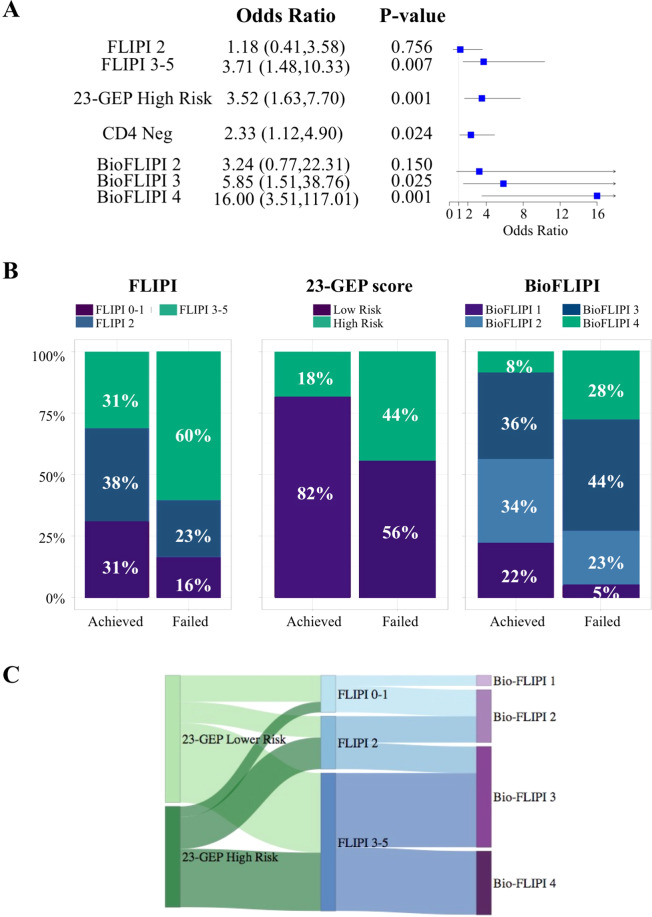
Intrafollicular CD4 + expression and tumor gene expression profile are independent prognostic factors in follicular lymphoma. **A** Forest plots showing the association between FLIPI, 23-GEP, and CD4 + expression and BioFLIPI risk groups and early failure. **B** Bar graph showing the percentages of 152 follicular lymphoma patients treated with immunochemotherapy who achieved or not event-free survival at 24 months in the indicated risk groups of FLIPI, 23-GEP, and BioFLIPI risk models. **C** Distribution of the follicular lymphoma patients from (**B**) who failed EFS24 (*n* = 43) from the FLIPI subgroups (center) within the 23-GEP (left) and BioFLIPI (right) risk groups.

Since the microenvironment may be influenced by the genomic composition of tumor, we next investigated whether the CD4 + expression and BioFLIPI were impacted by genetic features of the tumor, as assessed by the validated 23-gene expression profiling panel (23-GEP) [7]. Of the 186 FL cases treated with IC, 152 had digital expression quantification of the 23-GEP (Supplementary Table 9). Twenty-eight percent of these patients failed to achieve EFS24. Lack of CD4 + intrafollicular expression (38% of patients, OR 2.33; 95% CI 1.12–4.90; *p* = 0.024) and high-risk 23-GEP score (26% of patients, OR 3.52, 95% CI 1.63–7.70; *p* = 0.001) each predicted early failure (Fig. 6A), and in a multivariable model that included FLIPI, both CD4 + expression (OR 2.26, 95% CI 1.02–5.07; *p* = 0.046) and the 23-GEP score (OR 2.26, 95% CI 0.97–5.25; *p* = 0.057) remained predictors. Similarly, the BioFLIPI modeled as a continuous score (1–4, OR per one-point increase 2.31, 95% CI 1.51–3.68; *p* < 0.001) predicted early failure, and the association remained (OR 2.14, 95% CI 1.37–3.46; *p* < 0.001) when the high-risk 23-GEP score (OR 2.79, 95% CI 1.24–6.31; *p* = 0.013) was included in the model. When stratified on 23-GEP score, BioFLIPI was a stronger predictor of early failure in low-risk (74%, OR 2.51, 95% CI 45–4.70; *p* = 0.002) relative to high-risk (26%, OR 1.55, 0.73–3.52; *p* = 0.27) patients, supporting their independent prognostic value. Similar patterns were observed for EFS and OS. Among the analyzed genes, only three differed significantly in patients who failed EFS24 and lack of intrafollicular CD4 + expression: USP44, E2FS, and EML6 (Supplementary Table 10). These are BCL6-target genes [23] with a critical role in B-cell terminal differentiation and proliferation [24–26]. Aberrant expression of these genes leads to dysregulation of GC-specific transcription signatures by a direct or indirect effect as they can bind to the DNA, stabilize, or be recruited by chromatin modifiers as part of the transcriptional machinery [24–30]. However, whether USP44, E2FS, and EML6 may play a role in regulating tumor-immune interaction remains to be determined. Taken together, our data suggest that the CD4 + T-cell infiltrate and tumor gene expression are independently predictive of outcome in FL, and future research should consider functional studies to elucidate their mutual influence.

Finally, we assessed the performance of the three prognostic models to predict early failure in our cohort of patients. The sensitivity (the true positive rate) of the BioFLIPI 3–4 (72%) to identify early failure was superior than FLIPI 3–5 (60%) and high-risk 23-GEP score (44%). However, 44% of patients who achieved EFS24 were still assigned into the high-risk BioFLIPI subgroups compared with 31% of FLIPI 3–5 and 18% of 23-GEP score high risk. (Fig. 6B) We then explored patients’ redistribution among the three risk models to identify differences and overlaps. Among patients who failed EFS24, the BioFLIPI 3–4 captured all high-risk and 50% of intermediate FLIPI groups, while 23-GEP score high-risk group included 40% of FLIPI 3–5, 60% of FLIPI 2, and about 10% of FLIPI 0–1 (Fig. 6C). These data further support the conclusion that BioFLIPI and the 23-GEP score are complementary risk models and their combination will likely maximize identification of patients at risk of early failure.

## Discussion

Disease heterogeneity and lack of understanding of disease mechanisms are major hindrances to identifying patients with follicular lymphoma at risk for early clinical failure. To improve upon this, we investigated the lymphoma microenvironment and found that the lack of intrafollicular CD4 + memory T cells plays a critical role in treatment failure. We have leveraged the characterization of this prognostic immune biomarker to define a novel bio-clinical risk model, termed BioFLIPI, which may have major implications for therapeutic decision-making. The BioFLIPI utilizes a simple and commonly available IHC analysis, which however will require independent validation.

This study stems from the observation that a significant proportion of patients with FL experience an early relapse and a subsequent poor outcome. Thus, there is an urgent need to identify baseline features that can be used to define the prognostic profile early in the course of the disease. The FLIPI score has been developed as a clinical prognostic tool, but it does not predict disease behavior [31]. A first attempt to improve the prognostic assessment was the m7-FLIPI, a clinic–genetic risk model that integrates the mutational status of seven genes (EZH2, ARID1A, MEF2B, EP300, FOX01, CREBBP, and CARD11) with the FLIPI [6]. Similarly, the 23-gene predictor uses the expression levels of 23 genes associated with a risk of progression but not OS [7]. The m7-FLIPI and the 23-gene model were important steps forward in identifying high-risk patients, however, both are more complicated to implement widely due to the need for sequencing or digital gene expression profiling.

Recently, a plethora of studies have addressed the complex relationship between FL B cells and the tumor microenvironment within the follicular malignant niche, but no definitive conclusion has been reached regarding the specific cell type or location of the cells responsible for the impact on patient outcome [13, 32–35]. Recently, PD-L2 expression was identified as a promising prognostic biomarker and noted to associate with low tumor-infiltrating immune cells (including CD4 + ); however, the cellular phenotype and the spatial discrimination between intra- and perifollicular localization were not investigated [36]. Another study reported the association between early transformation and active intrafollicular CD4 + T cells [12], supporting a functional role of this cell population. Here, we demonstrated the prognostic value of central memory CD4 + T cells specifically located within the follicle in close proximity to other T cells. In particular, lack of intrafollicular CD4 + expression was strongly associated with failing to achieve EFS12/24 in patients who received or not immunochemotherapy. Remarkably, the correlation between CD4 + expression and outcome was confirmed with both IHC and CyTOF. However, unlike prior studies [37,38,], the prognostic significance of our model was lost when adjusting for chemotherapy backbones probably due to the low power of each subgroup. To the best of our knowledge, this is the first report about the critical role of intrafollicular, rather than total [39,40,] CD4 + expression in predicting early failure and outcome in follicular lymphoma patients.

The intrafollicular CD4 + T-cell population associated with favorable outcome was immunophenotypically characterized as activated, non-exhausted central memory T-cells. Previous studies showed that the T_CM_ cell phenotype plays a critical role in the immune surveillance of peripheral tissues [41]. In particular, the T_CM_ CD26 + is a subset recently characterized for its stemness and antitumor immunity [22]. Lack of the activated T_CM_ cells may disrupt the immune surveillance, enabling immune escape, which in turn allows FL B-cells to persist, facilitating lymphoid proliferation and transformation. Tumors accordingly manifest more aggressive features [42], thus providing the rationale for considering CD4 + activated T_CM_ cells as an independent prognostic biomarker. However, confirmatory functional studies will be warranted to validate the nature of these CD4 + T cells. Of note, CD4 + PD-L1 + T cells were not assessed in our panel. Given their role in the negative control of anti-tumor immunity [43], future investigation will be needed to assess PD-L1 + expression on intrafollicular CD4 + T cells. On the contrary, intratumoral T_REG_ cells were not prognostic in this study, despite their prevalence in lymphoma and their ability to suppress T-cell function [35,44,]. Similarly, T_FH_ cells were not associated with EFS. Even monocytic/macrophage cells did not show prognostic impact. In line with prior studies we observed a low to negligible number of intratumoral CD14 + cells; however, we did not confirm a correlation between SIRPα expression and survival [45]. This discrepancy might be due to the difference in the biomarker panel and the frequent loss of CD14 in FL tissue with concordant need of multiple markers (e.g., CD32 + and SIRPα) to identify macrophage population.

By adding the intrafollicular CD4 + expression to the well-established clinical risk model FLIPI, we developed an improved prognostic algorithm that can help to guide therapeutic decisions for newly diagnosed FL patients. Favorable-risk groups showed an indolent disease course and these patients therefore might be observed or receive lower-intensity approaches, while high-risk groups had an early relapse that suggests the need for a more intensive treatment or for clinical trials with novel agents and consideration for maintenance therapy. The BioFLIPI demonstrated a superior prognostic value than the FLIPI, confirming that both clinical and biological factors affect the outcome and should both be considered to provide optimal prognostic information. However, 44% of the two Bio-FLIPI high risk groups still achieve EFS24, suggesting that additional events influence the distinct biological behavior. While our model underlines the importance of tumor microenvironment, we reason that it may be influenced by the genetic composition of tumor. For example, CREBBP mutation, which occurs in about 60% of FL [46], has been associated with reduced expression of antigen presentation machinery, impaired immune surveillance, and inferior outcome as compared with CREBBP WT lymphoma patients [47]. It is possible that CREBBP mutations might lead to a lack of intrafollicular CD4 + expression as part of the same immune-escape mechanism resulting from aberrant transcription and translation of MHC class II molecules. Using 23 gene-expression profiling, we found the aberrant expression of USP44, E2FS, and EML6 in patients who failed EFS24 and lack of intrafollicular CD4 + cells. These are BCL-6 target genes [23] that control transcriptional programs involved in B-cell terminal differentiation and proliferation by direct DNA binding, as part of transcriptional complexes or through their stabilization [24–30]. Aberrant expression of USP44, E2FS, and EML6 can be due to mutations of their genes or secondary to dysregulation of transcription factors and chromatin modifiers that control or functionally balance BCL6, such as CREBBP and EZH2. This latter scenario is intriguing since it might imply a link with immune evasion through already-known mechanisms [46–48]. However, mechanistic studies are warranted to elucidate how aberrations in these newly identified genes associate with dysregulation in lymphoma microenvironment. Remarkably, we found that CD4 + expression, BioFLIPI, and 23-GEP scores are independently prognostic. This highlights that the tumor genetic features and the CD4 + T-cell infiltration are both factors of paramount relevance for the outcome of FL patients. Future studies will be needed to standardize the assessment of intrafollicular CD4 + expression and validate the prognostic value of the BioFLIPI risk model alone and in combination with tumor genetic features in independent patient cohorts.

From a clinical perspective, our data provide a rationale for selecting a therapeutic approach aimed at restoring the ability of tumor-infiltrating lymphocytes to recognize and kill lymphoma cells in high-risk BioFLIPI patients who are less likely to respond to conventional immunochemotherapy. We propose that lack of intrafollicular CD4 + T cells can be considered as a surrogate biomarker of immune escape in a manner analogous to the case of CREBBP mutant lymphomas, which indeed benefit from HDAC3-selective inhibitors to promote immune-related activities [47]. Additional studies will be needed to see whether the BioFLIPI predicts responses to immunotherapy and whether it identifies patients who may require more intensive chemoimmunotherapy approaches.

In conclusion, the BioFLIPI represents a promising predictor of treatment outcome in newly diagnosed FL patients that incorporates biological and clinical features, but that will need independent validation and eventually combination with the genomic landscape. While additional investigation to determine the mechanisms behind the reduced or absent expression of CD4 + T cells inside the lymphoma follicles is warranted, the prognostic power of the BioFLIPI will be useful in the design of clinical trials as it identifies patients at the highest risk of early failure who may benefit most from more intensive therapies or novel frontline regimens.

## Supplementary information

Supplementary Material

Supplementary Figures
