# Supplementary material for: Lack of intrafollicular memory CD4 + T cells is predictive of early clinical failure in newly diagnosed follicular lymphoma
Source: Blood Cancer J. 2021 Jul 15;11(7):130. doi: 10.1038/s41408-021-00521-4 (PMC8282842; doi:10.1038/s41408-021-00521-4)

Mondello et al., Supplementary Material

**Lack of Intrafollicular Memory CD4+ T-cells is Predictive of**

**Early Clinical Failure in Newly Diagnosed Follicular Lymphoma**

Contents

Supplementary Methods

Supplementary Tables

Supplementary Table 1: Patient characteristics in the study and excluded cohorts.

Supplementary Table 2: Patient characteristics in the discovery and validation cohorts

Supplementary Table 3: Biomarkers expression in the discovery, validation and pooled cohorts of follicular lymphoma patients.

Supplementary Table 4: Biomarkers expression inside and outside the follicles of the discovery cohort which did not associate with early failure.

Supplementary Table 5: Reclassification of follicular lymphoma patients from FLIPI to BioFLIPI risk model.

Supplementary Table 6: Cox regression analysis on FLIPI and BioFLIPI risk groups associated with early event.

Supplementary Table 7: List of the antibodies used for the CyTOF experiment.

Supplementary Table 8: List of the nucleotide-barcoded primary antibodies used for the CODEX experiment.

Supplementary Table 9: Clinical characteristics of FL patients assesses with 23-GEP score.

Supplementary Table 10: Differentially expressed genes as per 23-GEP.

**SUPPLEMENTARY MATERIALS AND METHODS**

**Immunohistochemistry.**

Staining was performed on the TMA from diagnostic biopsies using the following antibodies: CD4 (#NCL-L-CD4-368, Leica), CD8 (#M7103, DAKO), FOXP3 (#14-4777-82, eBioscience), CD32b (#ab45143, Abcam), CD14 (#M0825, DAKO), CD68 (#M0876, DAKO), CD70 (#LS-C134544, Lifespan Bioscience), SIRPα (#ab53721, Abcam), TIM3 (#AF2365, R&D), PD-1 (#ab52587, Abcam) and PD-L1 (#ab58810, Abcam), but not PD-L2. These immunohistochemistry markers were selected to identify previously established immune populations with relevant prognostic value in follicular lymphoma including regulatory T (T_REG_)-cells, effector or exhausted T-cells, and suppressive monocytes/macrophages. Stains were scored to the nearest decile as the percentages of cells with positive staining for each marker inside and outside of the neoplastic follicles. Scores for each stain were visually evaluated and dichotomized as <10% vs ≥10% except for CD32b (<20% vs ≥20%) and SIRPα (<30% vs ≥30%). Staining inside and outside the follicle was scored separately. These cutoffs were made based on the distribution of scores and before assessment of outcome.

**Mass cytometry (CyTOF).**

The CyTOF assay was performed according to the manufacturer’s instructions. Briefly, 3 × 10^6^ cells were stained with 5 μM Cell-ID™ [Cisplatin](https://www.sciencedirect.com/topics/biochemistry-genetics-and-molecular-biology/cisplatin) (Fluidigm, San Francisco, CA) for 5 minutes and quenched with MaxPal Cell Staining Buffer (Fluidigm) using 5 times the volume of the cell suspension. After centrifugation, cell suspensions (50 μl) were incubated with 5 μL of human [Fc-receptor](https://www.sciencedirect.com/topics/immunology-and-microbiology/fc-receptor) Blocking solution (Biolegend, San Diego, CA) for 10 minutes and 50 μL of pre-mixed antibody cocktail for 30 minutes (Supplementary Table 7). After washing, cells were incubated with 1ml of cell intercalation solution (125nM MaxPal Intercalator-Ir into 1ml MaxPal Fix and Pem Buffer, Fluidigm) overnight at 4°C. Cells were centrifuged with MaxPal Water and pelleted. The pelleted cells were suspended with EQ Calibration Beads (Fluidigm) and cell events were acquired by a CyTOF II instrument (Fluidigm).

**CyTOF data analysis.**

The CyTOF data were analyzed using online Cytobank software.^1^ All the samples were normalized and analyzed simultaneously to account for variability in signal across long acquisition times. Linage markers (CD4, [CD8](https://www.sciencedirect.com/topics/neuroscience/cd8), TIM3, PD-1, [CXCR5](https://www.sciencedirect.com/topics/immunology-and-microbiology/cxcr5), CCR7, CD45RO, ICOS, TIGIT or BTLA) were used to separate T-cells into T-reg, naïve, follicular helper, memory, effector or exhausted subsets. SIRPα and CD14 were used to identify monocytes and macrophages. All the antibodies were purchased from Fluidigm We used the CITRUS method^2^ to first perform hierarchical cluster analysis on the immune cell subsets using Euclidean distance as dissimilarity measure and Ward’s linkage as agglomeration method, and then assess clinical relevance by performing supervised penalized classification to identify cluster features that are associated with EFS12/24.

**CODEX tissue preparation.**

An 8-micron thick section was obtained from formalin-fixed paraffin-embedded (FFPE) tissue block containing a lymph node specimen from follicular lymphoma patients. The section was mounted on a poly-lysine coated glass cover slip and stained with a cocktail containing 15 nucleotide-barcoded primary antibodies following heat-based antigen retrieval with histone H3 as control (Supplementary Table 8). Tissue was stained using the following antibodies purchased from Akoya: DAPI (#7000003), CD31-BX001 (EP3095)—Alexa Fluor™ 750-RX001 (#4450017), CD20-BX007 (L26)—Alexa Fluor™ 750-RX007 (#4450018), Pan-Cytokeratin-BX019 (AE-1/AE-3)—Alexa Fluor™ 750-RX019 (#4450020), CD3e-BX045(EP449E)—Cy5-RX045 (#4450030), CD44-BX005 (IM7)—Atto 550-RX005 (#4250002), E-cadherin-BX014 (4A2C7)—Atto 550-RX014 (#4250021), CD45RO-BX017 (UCHL1)—Atto 550-RX017 (#4250023), CD8-BX026 (C8/144B)—Atto 550-RX026 (#4250012), Ki67-BX047 (B56)—Atto 550-RX047 (#4250019), CD4-BX003 (EPR6855)—Cy5-RX003 (#4350018), CD107a-BX006 (H4A3)—Cy5-RX006 (#4350001), CD68-BX015 (KP1)—Cy5-RX015 (#4350019), CD8-BX026 (C8/144B)—Atto 550-RX026 (#4250012), CD11c-BX024 (118/A5)—Cy5-RX024 (#4350020), Histone H3 Phospho(Ser28)-BX030 (HTA28)—Cy5-RX030 (#4350021), CD45-BX021(D9M81)—CY5-RX021 (#4450042). The section underwent nuclear staining (DAPI) and was loaded on the stage of an automated inverted fluorescence microscope connected to the robotic fluidic system known as Co-Detection by Indexing (CODEX, Akoya Biosciences). CODEX uses fluorophore-tagged complementary nucleotide sequences (reporters) to reveal three antibodies at a time per cycle in addition to the nuclear stain. Following imaging of the tissue, reporters are removed through an isothermic de-hybridization reaction. Tissue underwent a total of 8 iterative cycles including 2 blank cycles (beginning and end) for auto-fluorescence background subtraction.

**CODEX data analysis.**

An adjacent tissue section from the same FFPE block was stained with hematoxylin and eosin (H&E) and annotated by an expert pathologist to identify malignant follicles (black circles) and inter-follicular tumor areas (green circles) (Supplementary Fig. 7A). Using a 2X air objective, a panorama of the whole tissue section stained for CODEX was imaged using the DAPI channel. A total of five regions of interest (ROIs) were selected for imaging using a 20X air objective during the CODEX cycles (Supplementary Fig. 7B). Each ROI was further divided in follicular (F) and inter-follicular (IF) regions (Supplementary Fig. 7C-D). ROI size consisted of five by five tiles with 30% overlap. Following CODEX acquisition, images were processed using the CODEX processor software (Akoya Biosciences).

**Image processing and cells segmentation.**

Cell segmentation was performed using Unet semantic segmentation neural network with custom encoder, which was trained on manually segmented cells. Segmentation was based on nuclei (DAPI) and membrane (CD45) marker expression and allowed finding an area mask per each cell. The cell segmentation mask had an accuracy of approximately 80% (Supplementary Fig. 7E). For each cellular mask, the mean marker expression was measured within the borders. The masks for endothelial cells were generated using custom endothelium segmentation convolutional neural net with Unet-like architecture, based on CD31 expression. Cell typing was performed after scaling of markers expression and clustering using Phenograph (http://dx.doi.org/10.1016/j.cell.2015.05.047). Each obtained cluster of cells was named based on the expressions of the prevalent markers. The cell subtypes assignment was guided by clustering heatmaps and tSNE projections. Relative and absolute proportions of types were calculated for each region, and results represented as a bar plot. To visualize the cellular distribution on the ROI, for each region, the defined individual cellular masks were colored according to its cell type using opencv2 library. The density of the distribution of the cells across the region was visualized with a random Voronoi tessellation of the slide, based on the method described by Yuan^3^. Bright color represents a higher density. All plots were made using matplotlib, seaborn and opencv2 python libraries. The follicles were manually annotated for analysis.

**Cellular contacts and communities analysis.**

Cellular contacts were measured as a number and types of contacting cellular masks to the current cellular mask within two cellular diameters. The number of intersecting pixels with endothelium mask within the two cellular diameters around the cell was taken a measure of contacts with endothelial cells. To identify cellular communities, we clustered cells based on the number and types of neighboring masks using Phenograph. To do that, the number of cellular contacts was additionally scaled. Each identified community cluster was named based on the dominant neighboring cell type which defines it. For example, “CD4 memory community” is defined by the fact that members of this community demonstrate the highest number of contacts with CD4+ memory cells among all the other clustered communities.

**Comparative community analysis between intrafollicular and interfollicular regions.**

Intrafollicular regions on the fluorescence images were manually masked based on annotations provided by a pathologist on an adjacent H&E section. Cells with its center found within a mask were determined as Intrafollicular. The number of cell types and the number of cellular communities these cells represent were calculated for regions within the follicles and outside the follicles. Since there were significantly fewer cells in the Intrafollicular regions than outside them, for a more accurate comparison we randomly selected regions from outside the follicle with the total area equal to the area of the intrafollicular mask. The significance (non-randomness) of cellular interactions or contacts was measured using a permutation test as previously described by Schapiro et al.^4^ Cell types were randomly permuted 100,000 times, and the percentages of cellular contacts in random permutations were compared with observed contacts. The significance of a neighboring interaction between each pair of cell types is visualized as a heatmap in which rows represent the neighborhood of a cell phenotype of interest and columns the enrichment cell in other neighborhoods. The final heatmap represents p-values of each type of neighboring.

Supplementary References

1. Kotecha N, Krutzik PO, Irish JM. Web-based analysis and publication of flow cytometry experiments. *Curr. Protoc. Cytom.* 2010;Chapter 10(1):Unit10.17.

2. Bruggner R V., Bodenmiller B, Dill DL, Tibshirani RJ, Nolan GP. Automated identification of stratifying signatures in cellular subpopulations. *Proc. Natl. Acad. Sci. U. S. A.* 2014;111(26):.

3. Yuan Y. Spatial heterogeneity in the tumor microenvironment. *Cold Spring Harb. Perspect. Med.* 2016;6(8):.

4. Schapiro D, Jackson HW, Raghuraman S, et al. HistoCAT: Analysis of cell phenotypes and interactions in multiplex image cytometry data. *Nat. Methods*. 2017;14(9):873–876.

| **Supplementary Table 1.** Patient characteristics in the study and excluded cohorts. | | | |
| --- | --- | --- | --- |
|  | **In study (n=496)** | **Not in Study (n=422)** | *P*-value |
| **Age (years)** |  |  |  |
| mean (SD) | 58.6 (13.1) | 60.3 (13.2) | 0.06 |
| median | 59 | 61 |  |
| range | 23.0 – 93.0 | 19.0 – 88.0 |  |
| **Gender** |  |  | 0.91 |
| female | 237 (47.8%) | 200 (47.4%) |  |
| male | 259 (52.2%) | 222 (52.6%) |  |
| **PS Group** |  |  | 0.53 |
| <2 | 473 (95.4%) | 405 (96.2%) |  |
| ≥2  missing | 23 (4.6%)  0 | 16 (3.8%)  1 |  |
| **Ann Arbor Stage** |  |  | 0.06 |
| I-II | 147 (29.6%) | 149 (35.3%) |  |
| III-IV  missing | 349 (70.4%)  0 (0%) | 272 (64.5%)  1 (0.2%) |  |
| **LDH** |  |  | 0.05 |
| normal | 333 (76.6%) | 301 (82.2%) |  |
| > ULN  missing | 102 (23.4%)  61 | 65 (17.8%)  56 |  |
| **Hemoglobin** |  |  | 0.76 |
| ≥12 g/dL | 404 (87.1%) | 337 (87.8%) |  |
| <12 g/dL  missing | 60 (12.9%)  32 | 47 (12.2%)  38 |  |
| **Nodal involvement** |  |  | 0.002 |
| ≤4 | 294 (60.2%) | 286 (70.3%) |  |
| >4  missing | 194 (39.8%)  8 | 121 (29.7%)  15 |  |
| **Grade** |  |  | 0.002 |
| 1-2 | 417 (84.1%) | 384 (91.0%) |  |
| 3a | 79 (15.9%) | 38 (9.0%) |  |
| **FLIPI** |  |  | 0.14 |
| 0  1  2  3  4  5 | 59 (11.9%)  125 (25.2%)  179 (36.1%)  98 (19.8%)  29 (5.8%)  6 (1.2%) | 50 (11.8%)  141 (33.4%)  135 (32.0%)  72 (17.1%)  21 (5.0%)  3 (0.7%) |  |
| **Initial Treatment** |  |  | 0.34 |
| IC | 199 (40.1%) | 149 (35.3%) |  |
| Observation | 171 (34.5%) | 155 (36.7%) |  |
| other | 35 (7.1%) | 38 (9.0%) |  |
| R monotherapy  Surgery only | 57 (11.5%)  0 (0.0%) | 53 (12.6%)  2 (0.5%) |  |
| XRT only | 34 (6.9%) | 25 (5.9%) |  |
| **OS**  events (N)  median survival (years)  median follow-up (years)  **Primary COD**  Lymphoma-related  Therapy-infection  Therapy-cardiac  Secondary malignancy  Bleomycin-induced lung injury  other causes  unable to obtained records  missing  **EFS**  events (N)  median survival (years)  median follow-up (years)  **Achieved EFS12/24** | 110  NA  8.1  44 (42.7%)  8 (7.8%)  3 (2.9%)  18 (17.5%)  1 (1.0%)  16 (15.6%)  13 (12.6%)  393  279  5.5  8.0 | 79  NA  8.0  27 (35.5%)  5 (6.6%)  2 (2.6%)  13 (17.1%)  0 (0.0%)  16 (20.7%)  13 (17.1%)  346  220  6.1  7.0 | 0.27  0.79  0.42  0.33 |
| achieved | 388 (78.2%) | 334 (80.9%) |  |
| failed | 108 (21.8%) | 79 (19.1%) |  |
| missing | 0 | 9 |  |

**Abbreviations**: n, number; SD, standard deviation; PS, performance status; LDH, lactate dehydrogenase; ULN, upper limit of normal; FLIPI, Follicular Lymphoma International Prognostic Index; IC, immunochemotherapy; R, rituximab; XRT, radiotherapy; FU, follow up; OS, overall survival; NA, not applicable; COD, cause of death; EFS, event free survival.

| **Supplementary Table 2.** Patient characteristics in the discovery and validation cohorts. | | | |
| --- | --- | --- | --- |
|  | **Discovery (n=166)** | **Validation (n=330)** | *P*-value |
| **Age (years)** |  |  |  |
| mean (SD) | 59.5 (14.0) | 58.2 (12.6) | 0.30 |
| median | 60 | 58 |  |
| range | 23.0 – 91.0 | 24.0 – 93.0 |  |
| >60 | 81 (48.8%) | 137 (41.5%) | 0.12 |
| **Gender** |  |  | 0.28 |
| female | 85 (51.2%) | 152 (46.1%) |  |
| male | 81 (48.8%) | 178 (53.9%) |  |
| **PS Group** |  |  | 0.09 |
| <2 | 162 (97.6%) | 311 (94.2%) |  |
| ≥2 | 4 (2.4%) | 19 (5.8%) |  |
| **Ann Arbor Stage** |  |  | 0.80 |
| I-II | 48 (28.9%) | 99 (30.0%) |  |
| III-IV | 118 (71.1%) | 231 (70.0%) |  |
| **LDH** |  |  | 0.19 |
| normal | 118 (80.3%) | 215 (74.7%) |  |
| > ULN | 29 (19.7%) | 73 (25.3%) |  |
| **Hemoglobin** |  |  | 0.99 |
| ≥12 g/dL | 135 (81.3%) | 269 (81.5%) |  |
| <12 g/dL | 20 (12.1%) | 40 (12.1%) |  |
| **Nodal involvement** |  |  | 0.53 |
| ≤4 | 102 (62.2%) | 192 (59.3%) |  |
| >4 | 62 (37.8%) | 132 (40.7%) |  |
| **Grade** |  |  | 0.69 |
| 1-2 | 138 (83.1%) | 279 (84.5%) |  |
| 3a | 28 (16.9%) | 51 (15.5%) |  |
| **FLIPI** |  |  | 0.52 |
| 0  1  2  3  4  5 | 20 (12.0%)  41 (24.7%)  58 (34.9%)  38 (22.9%)  6 (3.6%)  3 (1.8%) | 39 (11.8%)  84 (25.5%)  121 (36.7%)  60 (18.2%)  23 (7.0%)  3 (0.9%) |  |
| **Initial Treatment** |  |  | 0.049 |
| IC | 62 (37.3%) | 137 (41.5%) |  |
| Observation | 55 (33.1%) | 116 (35.2%) |  |
| other | 20 (12.0%) | 15 (4.5%) |  |
| R monotherapy | 18 (10.8%) | 39 (11.8%) |  |
| XRT only | 11 (6.6%) | 23 (7.0%) |  |
| **OS**  events (N)  median survival (years)  median follow-up (years)  **Primary COD** Lymphoma-related  Therapy-infection  Therapy-cardiac  Secondary malignancy  Bleomycin-induced  lung injury  other causes  unable to obtained  records  **EFS**  events (N)  median survival (years)  median follow-up (years)  **Achieved EFS12/24** | 46  14.4  11.3  17(39.5)  1 (2.3%)  1 (2.3%)  11 (25.6%)  0 (0%)  6 (14.0%)  7 (16.3%)  98  6.3  10.9 | 64  NA  6.9  27 (45.8%)  7 (11.9%)  2 (3.4%)  7 (11.9%)  1 (1.7%)  9 (15.3%)  6 (10.2%)  181  5.0  6.9 | 0.676  0.302  0.234  0.236 |
| achieved | 135 (81.3%) | 253 (76.7%) |  |
| failed | 31 (18.7%) | 77 (23.3%) |  |

**Abbreviations**: n, number; SD, standard deviation; PS, performance status; LDH, lactate dehydrogenase; ULN, upper limit of normal; FLIPI, Follicular Lymphoma International Prognostic Index; IC, immunochemotherapy; R, rituximab; XRT, radiotherapy; FU, follow up; OS, overall survival; NA, not applicable; COD, cause of death; EFS, event free survival.

**Supplementary Table 3.** Biomarkers expression in the discovery, validation, and pooled cohorts of follicular lymphoma patients.

| **Marker** | **Discovery**  **(n=166)** | | | | **Validation**  **(n=330)** | | | | **Pooled**  **(n=496)** | | | |
| --- | --- | --- | --- | --- | --- | --- | --- | --- | --- | --- | --- | --- |
|  | No Early Event | Early Event | OR  (95% CI) | P-value | No Early Event | Early Event | OR  (95% CI) | P-value | No Early Event | Early Event | OR  (95% CI) | P-value |
|  | N (%) | |  | | N (%) | |  | | N (%) | |  | |
| **CD4 inside** |  | | | | | | | | | | | |
| <10 | 48 (36.1) | 16 (50.0) | 1.77 (0.81,3.88) | 0.15 | 86 (36.1) | 41 (59.4) | 2.59 (1.50,4.51) | 0.001 | 134 (36.1) | 57 (56.4) | 2.29 (1.47,3.60) | 0.0002 |
| ≥10 | 85 (63.9) | 16 (50.0) | 1 (reference) |  | 152 (63.9) | 28 (40.6) | 1 (reference) |  | 237 (63.9) | 44 (43.6) | 1 (reference) |  |
| **CD4 outside** |  | | | | | | | | | | | |
| <10 | 45 (33.8) | 17 (53.1) | 2.22 (1.01,4.89) | 0.05 | 23 (9.7) | 10 (14.1) | 1.52 (0.66,3.28) | 0.30 | 68 (18.4) | 27 (26.2) | 1.57 (0.93,2.60) | 0.08 |
| ≥10 | 88 (66.2) | 15 (46.9) | 1 (reference) |  | 213 (90.3) | 61 (85.9) | 1 (reference) |  | 301 (81.6) | 76 (73.8) | 1 (reference) |  |
| **CD8 inside** |  | | | | | | | | | | | |
| <10 | 49 (36.6) | 17 (53.1) | 1.97 (0.90,4.33) | 0.09 | 119 (49.4) | 39 (57.4) | 1.38 (0.80,2.39) | 0.25 | 168 (44.8) | 56 (56.0) | 1.57 (1.01,2.45) | 0.05 |
| ≥10 | 85 (63.4) | 15 (46.9) | 1 (reference) |  | 122 (50.6) | 29 (42.6) | 1 (reference) |  | 207 (55.2) | 44 (44.0) | 1 (reference) |  |
| **FoxP3 outside** |  | | | | | | | | | | | |
| <10 | 80 (60.2) | 13 (40.6) | 0.45 (0.20,0.99) | 0.05 | 64 (26.1) | 23 (31.1) | 1.28 (0.71,2.24) | 0.4 | 144 (38.1) | 36 (34.0) | 0.84 (0.53,1.31) | 0.44 |
| ≥10 | 53 (39.8) | 19 (59.4) | 1 (reference) |  | 181 (73.9) | 51 (68.9) | 1 (reference) |  | 234 (61.9) | 70 (66.0) | 1 (reference) |  |
| **PD-1 inside** |  | | | | | | | | | | | |
| <10 | 48 (36.4) | 18 (56.2) | 2.25 (1.03,5.00) | 0.04 | 45 (19.7) | 13 (19.7) | 1.00 (0.49,1.94) | 0.99 | 93 (25.8) | 31 (31.6) | 1.33 (0.81,2.15) | 0.25 |
| ≥10 | 84 (63.6) | 14 (43.8) | 1 (reference) |  | 183 (80.3) | 53 (80.3) | 1 (reference) |  | 267 (74.2) | 67 (68.4) | 1 (reference) |  |
| **SIRPα inside** |  | | | | | | | | | | | |
| <30 | 114 (87.7) | 22 (73.3) | 0.39 (0.15,1.05) | 0.05 | 231 (97.9) | 65 (97.0) | 0.70 (0.15,4.99) | 0.68 | 345 (94.3) | 87 (89.7) | 0.53 (0.25,1.21) | 0.11 |
| ≥30 | 16 (12.3) | 8 (26.7) | 1 (reference) |  | 5 (2.1) | 2 (3.0) | 1 (reference) |  | 21 (5.7) | 10 (10.3) | 1 (reference) |  |

**Abbreviations**: N, number; OR, Odds Ratios; CI, confidence intervals; CD, cluster of differentiation; FOXP3, forkhead box protein 3; PD-1, programmed cell death protein 1; SIRPα, signal-regulatory protein alpha.

| **Supplementary Table 4.** Biomarkers expression inside and outside the follicles of the discovery cohort which did not associate with early failure. | | | | | | |
| --- | --- | --- | --- | --- | --- | --- |
| **Marker** | No early event | |  | Early event | |  |
|  | N (%) | |  | N (%) | | P-value |
| **CD8 outside** |  |  |  |  |  | 0.87 |
| <10 | 5 (3.7) | |  | 1 (3.1) | |  |
| ≥10 | 129 (96.3) | |  | 31 (96.9) | |  |
| **CD14 Inside** |  |  |  |  |  | 0.82 |
| <10 | 106 (81.5) | |  | 25 (83.3) | |  |
| ≥10 | 24 (18.5) | |  | 5 (16.7) | |  |
| **CD14 Outside** |  |  |  |  |  | 0.69 |
| <10 | 95 (73.1) | |  | 23 (76.7) | |  |
| ≥10 | 35 (26.9) | |  | 7 (23.3) | |  |
| **CD32b Inside** |  |  |  |  |  | 0.72 |
| <10 | 21 (16.2) | |  | 6 (18.8) | |  |
| ≥10 | 109 (83.8) | |  | 26 (81.3) | |  |
| **CD32b Outside** |  |  |  |  |  | 0.71 |
| <10 | 41 (31.5) | |  | 9 (28.1) | |  |
| ≥10 | 89 (68.5) | |  | 23 (71.9) | |  |
| **CD68 Inside** |  |  |  |  |  | 0.52 |
| 0 | 23 (17.2) | |  | 4 (12.5) | |  |
| ≥10 | 111 (82.8) | |  | 28 (87.5) | |  |
| **CD68 Outside** |  |  |  |  |  | 0.35 |
| <10 | 45 (33.6) | |  | 8 (25.0) | |  |
| ≥10 | 89 (66.4) | |  | 24 (75.0) | |  |
| **CD70 Inside** |  |  |  |  |  | 0.86 |
| <10 | 10 (8.1) | |  | 2 (7.1) | |  |
| ≥10 | 113 (91.9) | |  | 26 (92.9) | |  |
| **CD70 Outside** |  |  |  |  |  | 0.95 |
| <10 | 30 (24.4) | |  | 7 (25.0) | |  |
| ≥10 | 93 (75.6) | |  | 21 (75.0) | |  |
| **FoxP3 inside** |  |  |  |  |  | 0.34 |
| <10 | 87 (65.4) | |  | 18 (56.3) | |  |
| ≥10 | 46 (34.6) | |  | 14 (43.8) | |  |
| **PD-1 outside** |  |  |  |  |  | 0.60 |
| <10 | 106 (80.3) | |  | 27 (84.4) | |  |
| ≥10 | 26 (19.7) | |  | 5 (15.6) | |  |
| **PD-L1 INSIDE** |  | |  |  | | 0.58 |
| <10 | 83 (62.4) | |  | 21 (67.7) | |  |
| ≥10 | 26 (19.7) | |  | 10 (32.3) | |  |
| **PD-L1 OUTSIDE** |  | |  |  | | 0.51 |
| <10 | 73 (54.9) | |  | 15 (48.4) | |  |
| ≥10 | 60 (45.1) | |  | 16 (51.6) | |  |
| **SIRPα outside** |  |  |  |  |  | 0.21 |
| <30 | 115 (88.5) | |  | 29 (96.7) | |  |
| ≥30 | 15 (11.5) | |  | 1 (3.3) | |  |
| **TIM3 Inside** |  |  |  |  |  | 0.83 |
| <10 | 38 (30.6) | |  | 8 (28.6) | |  |
| ≥10 | 86 (69.4) | |  | 20 (71.4) | |  |
| **TIM3 Outside** |  |  |  |  |  | 0.78 |
| <10 | 25 (20.2) | |  | 5 (17.9) | |  |
| ≥10 | 99 (79.8) | |  | 23 (82.1) | |  |

**Abbreviations**: N, number; CD, cluster of differentiation; FOXP3, forkhead box protein 3; PD-1, programmed cell death protein 1; PD-L1 programmed death-ligand 1; SIRPα, signal-regulatory protein alpha; TIM3, T-cell immunoglobulin 3.

**Supplementary Table 5.** Reclassification of follicular lymphoma patients from FLIPI to BioFLIPI risk model.

| **FLIPI** | | **BioFLIPI** | |
| --- | --- | --- | --- |
| **Risk group** | Patients (N) | Patients (N) | Risk group |
| **Low Risk (0-1)** | 184  179  133 | 117  67  107  72  81  52 | **1** FLIPI 0-1 & CD4+ |
|  |  |  | **2** FLIPI 0-1 & CD4-  FLIPI 2 & CD4+ |
| **Intermediate Risk (2)** |  |  |  |
|  |  |  | **3** FLIPI 2 & CD4-  FLIPI 3-5 & CD4+ |
| **High Risk (3-5)** |  |  |  |
|  |  |  | **4** FLIPI 3-5 & CD4- |

CD4+ Intrafollicular; CD4- Intrafollicular.

**Supplementary Table 6.** Cox regression analysis on FLIPI and BioFLIPI risk groups associated with early event.

|  | **OR** | **95% CI** | **P-value** | **Concordance** |
| --- | --- | --- | --- | --- |
| **All cases (EFS12/24)** |  |  |  |  |
| FLIPI 0-1 | ref |  |  |  |
| FLIPI 2 | 1.80 | 1.03-3.18 | 0.04 | 0.636 |
| FLIPI 3-5 | 3.64 | 2.11-6.44 | < 0.001 |  |
|  |  |  |  |  |
| BioFLIPI 1 | ref |  |  |  |
| BioFLIPI 2 | 2.17 | 1.08-4.69 | 0.037 | 0.665 |
| BioFLIPI 3 | 3.53 | 1.78-7.54 | < 0.001 |  |
| BioFLIPI 4 | 8.92 | 4.00-21.08 | < 0.001 |  |
|  |  |  |  |  |
| **IC (EFS24)** |  |  |  |  |
| FLIPI 0-1 | ref |  |  |  |
| FLIPI 2 | 1.58 | 0.62-4.28 | 0.343 | 0.672 |
| FLIPI 3-5 | 4.86 | 2.11-12.34 | < 0.001 |  |
|  |  |  |  |  |
| BioFLIPI 1 | ref |  |  |  |
| BioFLIPI 2 | 2.77 | 0.82-12.78 | 0.133 | 0.695 |
| BioFLIPI 3 | 5.01 | 1.58-22.33 | 0.014 |  |
| BioFLIPI 4 | 15.11 | 4.19-73.61 | < 0.001 |  |
|  |  |  |  |  |
| **Non-IC (EFS12)** |  |  |  |  |
| FLIPI 0-1 | ref |  |  |  |
| FLIPI 2 | 1.91 | 0.96-3.87 | 0.067 | 0.582 |
| FLIPI 3-5 | 2.07 | 0.91-4.65 | 0.079 |  |
|  |  |  |  |  |
| BioFLIPI 1 | ref |  |  |  |
| BioFLIPI 2 | 1.91 | 0.82-4.86 | 0.150 | 0.620 |
| BioFLIPI 3 | 2.64 | 1.11-6.82 | 0.034 |  |
| BioFLIPI 4 | 4.93 | 1.59-15.55 | 0.005 |  |

**Abbreviations**: OR, Odds Ratios; CI, confidence intervals; HR, hazard ratio; EFS, event free survival; FLIPI, Follicular Lymphoma International Prognostic Index; ref, reference; IC, immunochemotherapy.

**Supplementary Table 7.** List of the antibodies used for the CyTOF experiment.

| **Vendor** | **Cat #** | **Target** | **Clone** | **Metal Tag** |
| --- | --- | --- | --- | --- |
| Fluidigm | 3089003B | CD45 | HI30 | 89Y |
| Fluidigm | 3141003A | CD196 (CCR6) | G034E3 | 141Pr |
| Fluidigm | 3142009B | CXCR1 | 8F1/CXCR1 | 142Nd |
| Fluidigm | 3143007B | CD5 | UCHT2 | 143Nd |
| Fluidigm | 3144007A | CD195 (CCR5) | NP-6G4 | 144Nd |
| Fluidigm | 3145001B | CD4 | RPA-T4 | 145Nd |
| Fluidigm | 3146001B | CD8a | RPA-T8 | 146Nd |
| Fluidigm | 3147006B | CD7 | CD76B7 | 147Sm |
| Fluidigm | 3148010B | CD14 | RMO52 | 148Nd |
| Fluidigm | 3149010B | CD25 (IL-2R) | 2A3 | 149Sm |
| Fluidigm | 3150016B | CD223 (LAG3) | 874501 | 150Nd |
| Fluidigm | 3151008B | CD278, ICOS | DX29 | 151Eu |
| Fluidigm | 3152010B | CD21 | BL13 | 152Sm |
| Fluidigm | 3153001B | TIGIT | MBSA43 | 153Eu |
| Fluidigm | 3154010B | TIM-3 | F38-2E2 | 154Sm |
| Fluidigm | 3155009B | CD279 (PD-1) | EH12.2H7 | 155Gd |
| Fluidigm | 3156004B | CD183 (CXCR3) | G025H7 | 156Gd |
| Fluidigm | 3158006A | CD194 (CCR4) | 205410 | 158Gd |
| Fluidigm | 3159003A | CD197 (CCR7) | G043H7 | 159Tb |
| Fluidigm | 3160003B | CD28 | CD28.2 | 160Gd |
| Fluidigm | 3161015B | CD26 | BA5B | 161DY |
| Fluidigm | 3162001B | CD69 | FN50 | 162Dy |
| Fluidigm | 3163009B | CD272/BTLA | MIH26 | 163Dy |
| Fluidigm | 3164009B | CD161 | HP-3G10 | 164Dy |
| Fluidigm | 3165025B | CD19 | HIB19 | 165Ho |
| Fluidigm | 3166017B | CD141 | M80 | 166Er |
| Fluidigm | 3167002B | CD27 | O323 | 167Er |
| Fluidigm | 3168017B | CD127 (IL-7R) | A019D5 | 168Er |
| Fluidigm | 3169008B | CD45RA | HI100 | 169Tm |
| Fluidigm | 3170001B | CD3 | UCHT1 | 170Er |
| Self-labeled | Self-labeled | KLRG1 | 2F1 | 171Yb |
| Fluidigm | 3172009B | CD57 | HCD57 | 172Yb |
| Fluidigm | 3173015B | 4-1BB/CD137 | 4B4-1 | 173Yb |
| Fluidigm | 3174001B | HLA-DR | L243 | 174Yb |
| Fluidigm | 3175024B | SIRPa/CD172a | SE5A5 | 175Lu |
| Fluidigm | 3176008B | CD56 (NCAM) | NCAM16.2 | 176Yb |
| Fluidigm | 3209002B | CD16 | 3GB | 209BI |

**Supplementary Table 8.** List of the nucleotide-barcoded primary antibodies used for the CODEX experiment.

|  | **Channel 1** | **Channel 2** | | **Channel 3** | | **Channel 4** | |
| --- | --- | --- | --- | --- | --- | --- | --- |
| **Cycle #** | DAPI Marker | AF750 | AF750 BX | Atto 550 | Atto 550 BX | Cy5 | Cy5 BX |
| **1** | DAPI-1 | CD31 | 1 | CD44 | 5 | CD4 | 3 |
| **2** | DAPI-2 | CD20 | 7 | E-Cad | 14 | CD107a | 6 |
| **3** | DAPI-3 | PanCK | 19 | CD45RO | 17 | CD68 | 15 |
| **4** | DAPI-4 | CD3 | 49 | CD8 | 26 | CD45 | 21 |
| **5** | DAPI-5 |  |  | Ki67 | 47 | CD11c | 24 |
| **6** | DAPI-6 |  |  |  |  | Histone H3 | 30 |
| **7** | DAPI-7 |  |  |  |  |  |  |
| **8** | DAPI-8 |  |  |  |  |  |  |

**Supplementary table 9.** Clinical characteristics of FL patients assesses with 23-GEP score.

|  |  | |
| --- | --- | --- |
|  | | **23-GEP subset (n=152)** |
| **Age (years)** | |  |
| mean (SD) | | 57.3 (12.3) |
| median | | 58.5 |
| range | | 24.0-87.0 |
|  | |  |
| **Gender** | |  |
| female | | 63 (41.5%) |
| male | | 89 (58.5%) |
| **PS Group** | |  |
| <2 | | 143 (94.1%) |
| ≥2 | | 9 (5.9%) |
| **Ann Arbor Stage** | |  |
| I-II | | 32 (21.0%) |
| III-IV | | 120 (79.0%) |
| **LDH** | |  |
| normal | | 89 (58,6%) |
| > ULN | | 43 (41.4%) |
| **Hemoglobin** | |  |
| ≥12 g/dL | | 115 (75.6%) |
| <12 g/dL | | 26 (24.4%) |
| **Nodal involvement** | |  |
| ≤4 | | 71 (46.7%) |
| >4 | | 80 (53.3%) |
| **Grade** | |  |
| 1-2 | | 100 (65.8%) |
| 3a | | 52 (34.2%) |
| **FLIPI** | |  |
| 0  1  2  3  4  5 | | 14 (9.2%)  27 (17.8%)  51 (33.6%)  42 (27.6%)  14 (9.2%)  4 (2.6%) |
| **OS**  events (N)  median survival (years)  median follow-up (years)  **Primary COD** Lymphoma-related  Therapy-infection  Therapy-cardiac  Secondary malignancy  Bleomycin-induced  lung injury  other causes  unable to obtained  records  **EFS**  events (N)  median survival (years)  median follow-up (years)  **Achieved EFS24** | | 33  NA  7.9  15 (45.5%)  4 (12.1%)  1 (3.0%)  4 (12.1%)  0 (0.0%)  6 (18.2%)  3 (9.1%)  72  10.5  7.9 |
| achieved | | 109 (71.7%) |
| failed | | 43 (28.3%) |
|  | |  |

**Abbreviations**: n, number; SD, standard deviation; PS, performance status; LDH, lactate dehydrogenase; ULN, upper limit of normal; FLIPI, Follicular Lymphoma International Prognostic Index; IC, immunochemotherapy; R, rituximab; XRT, radiotherapy; FU, follow up; OS, overall survival; NA, not applicable; COD, cause of death; EFS, event free survival.

**Supplementary Table 10.** Differentially expressed genes as per 23-GEP.


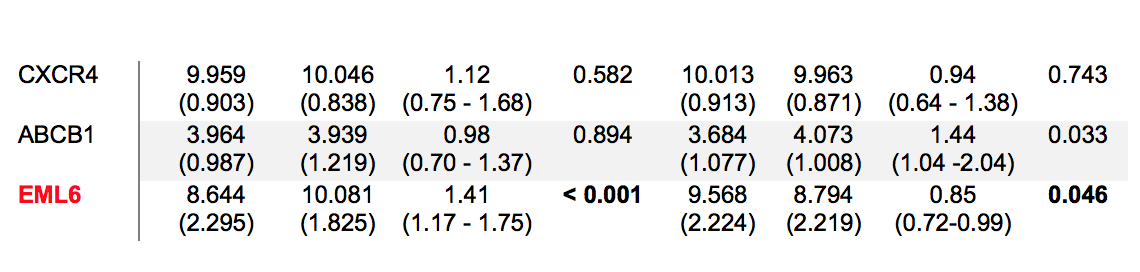

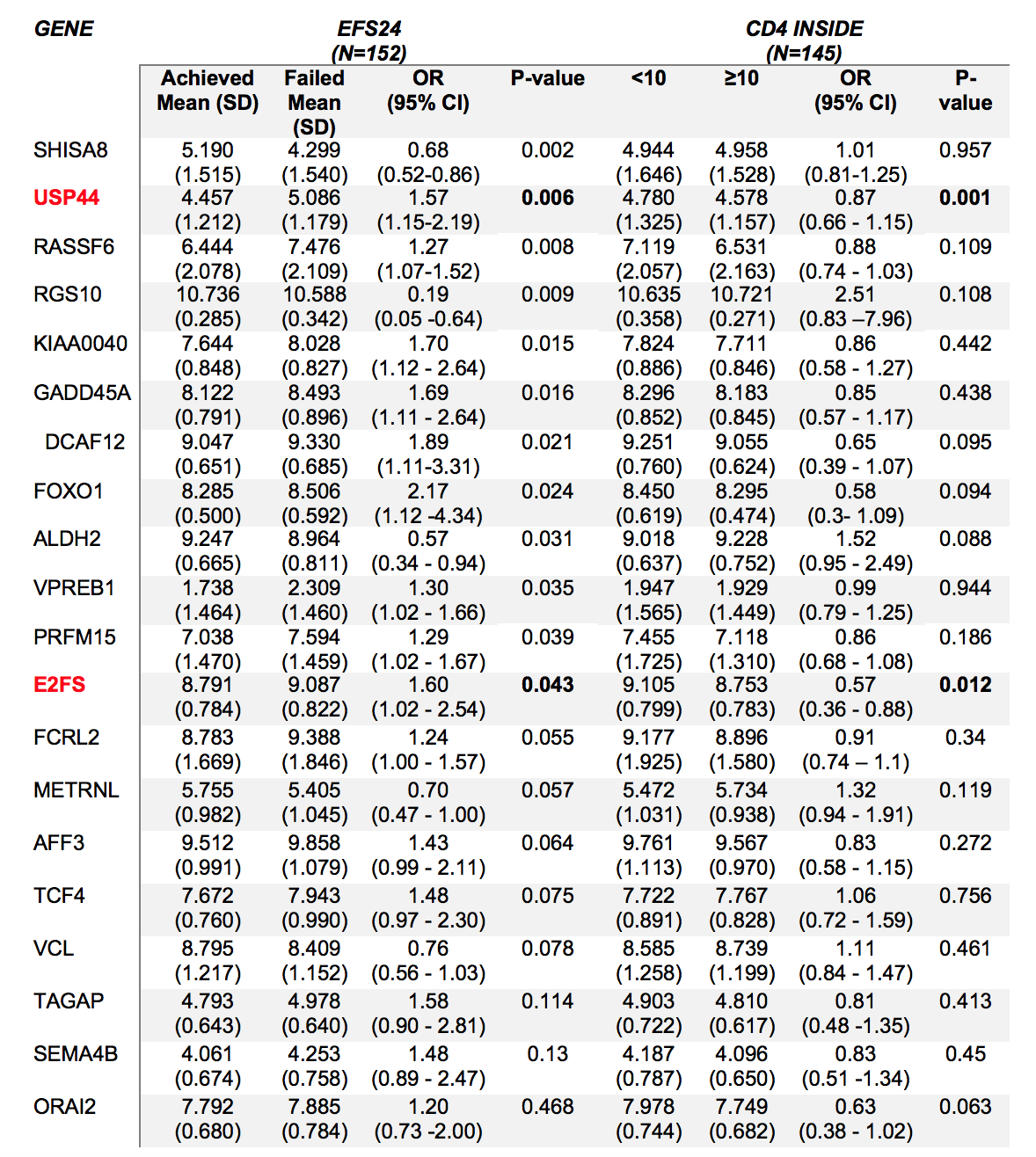

Supplement: Supplementary file 1 — Supplementary Material [file 41408_2021_521_MOESM1_ESM.doc]
