# Supplementary material for: Lack of intrafollicular memory CD4 + T cells is predictive of early clinical failure in newly diagnosed follicular lymphoma
Source: Blood Cancer J. 2021 Jul 15;11(7):130. doi: 10.1038/s41408-021-00521-4 (PMC8282842; doi:10.1038/s41408-021-00521-4)
Supplement: Supplementary file 2 — Supplementary Figures [file 41408_2021_521_MOESM2_ESM.doc]

Mondello et al., Supplementary Figures

**Lack of Intrafollicular Memory CD4+ T-cells is Predictive of**

**Early Clinical Failure in Newly Diagnosed Follicular Lymphoma**

Contents

Supplementary Fig. 1: consort diagram

Supplementary Fig. 2: Representative tissue microarray cores of Follicular Lymphoma stained for CD4, PD-1, FOXP3 and SIRPα.

Supplementary Fig. 3: Intrafollicular CD4+ expression is a prognostic immune-biomarker in Follicular Lymphoma.

Supplementary Fig. 4: Survival outcomes of patients with Follicular Lymphoma according to FLIPI score.

Supplementary Fig. 5: BioFLIPI improves risk stratification irrespective of treatment groups.

Supplementary Fig. 6: Mass cytometry analysis of Follicular Lymphoma patients.

Supplementary Fig. 7: Identification of follicular and inter-follicular areas.

Supplementary Fig. 8: CD8+ T-cells and naïve CD4+ T-cells are prevalent outside the follicles.

Supplementary Fig. 9: Central memory CD4+ T-cells are prevalent inside the follicles.

Supplementary Fig. 10: Minimal spanning tree (MST).

**Supplementary Fig. 1.** consort diagram

**Supplementary Fig. 2. Representative tissue microarray cores of Follicular Lymphoma**

**stained for CD4, PD-1, FOXP3 and SIRPα.**

**Supplementary Fig. 3. Intrafollicular CD4+ expression is a prognostic immune biomarker**

**in Follicular Lymphoma.** Forest plots showing association between indicated immune

biomarkers and early failure in the discovery **A** and validation **B** cohorts.

**Supplementary Fig. 4. Survival outcomes of patients with Follicular Lymphoma**

**according to FLIPI score.** Kaplan-Meier curves representing event free survival (**A**) and overall

survival (**B**) in follicular lymphoma patients classified by FLIPI risk groups.

**Supplementary Fig. 5. BioFLIPI improves risk stratification irrespective of treatment**

**groups.** Kaplan Meier curves representing event free survival in Follicular Lymphoma patients

treated with or without immunochemotherapy and classified by BioFLIPI (**A-B**) or by FLIPI (**C-D**)

risk groups.

**Supplementary Fig. 6. Mass cytometry analysis of Follicular Lymphoma patients.** tSNE analysis showing the expression levels of indicated markers in fifty-one newly diagnosed Follicular Lymphoma patients.

**Supplementary Fig. 7. Identification of follicular and inter-follicular areas. A.** and **B.** Two

adjacent tissue sections from the same FFPE block were used: **A** was stained with hematoxylin

and eosin (H&E) and annotated by expert pathologist to identify malignant follicles (black

circles) and inter-follicular tumor areas (green circles), **B** was stained for CODEX and imaged

using the DAPI channel. A total of five regions of interest (ROIs) were selected for imaging

using a 20X air objective during the CODEX cycles. **C.** Representative image of the ROI #5

showing 2 malignant follicular (F1-2) and 2 inter-follicular (IF1-2) areas. **D.** Representative

image showing high magnification of malignant follicle F2 from ROI #5 with CD20+ depicted in

blue and the DAPI nuclear stain in white. **E.** Representative images of correspondence between

composite fluorescence images and segmentation efficiency.

**Supplementary Fig. 8. CD8+ T-cells and naïve CD4+ T-cells are prevalent outside the follicles. A.** Representative images showing CD3+ CD8+ T-cells outside the follicles. **B.** Representative images showing CD3+ CD4+ CD45RO- T-cells outside the follicles. **C.** Representative images showing CD4+ Memory T-cells inside the follicle.

**Supplementary Fig. 9. Central memory CD4+ T-cells are prevalent inside the follicles. A.** Representation of cell contours from neural network showing cell types (top) and community (bottom) identification. **B.** Density plots of subpopulation analysis.

**Supplementary Fig. 10. Minimal spanning tree (MST).** MST showing the relationship among

the cell clusters and their localization.
